# Efficacy and efficiency of information retrieval of community family physicians at the point of care: exploring the associations with information and computer literacy

**DOI:** 10.5195/jmla.2023.1539

**Published:** 2023-07-10

**Authors:** Jumana Antoun, Jennifer Lapin, Dennis Beck

**Affiliations:** 1 ja46@aub.edu.lb, Professor of Famly Medicine, American University of Beirut, Beirut, Lebanon.; 2 jennifer.lapin@mail.waldenu.edu, Contributing Faculty, Richard W. Riley College of Education and Leadership, Walden University, Minneapolis, Minnesota, USA.; 3 denbeck1@gmail.com, Associate Professor of Educational Technology, University of Arkansas, Fayetteville, Arkansas, USA.

**Keywords:** Evidence-based medicine, information literacy, digital literacy, computer literacy, family physicians, Arab countries

## Abstract

**Objective:**

This study aimed to measure the association between the efficacy/efficiency of digital information retrieval among community family physicians at the point of care and information and computer literacy.

**Methods:**

This study is a part of a cross-sectional anonymous online survey-based study among community family physicians who reported no affiliation with an academic institution in eight Arab countries.

**Results:**

A total of 72 physicians were included. The mean total score for the information literacy scale was 59.8 out of 91 (SD = 11.4). The mean score was 29.3 (SD = 5.6) out of 55 on the computer literacy scale. A one-way ANOVA revealed a statistically significant association between information literacy and information retrieval efficacy (F (2,69) = 4.466, p = 0.015) and efficiency of information retrieval (F (2.69) = 4.563, p = 0.014). Computer literacy was not associated with information retrieval efficacy or efficiency.

**Conclusion:**

The information and computer literacy scores of community family physicians in eight Arab countries are average. Information literacy, rather than computer literacy, is positively associated with the efficacy and efficiency of information retrieval at the point of care. There is room for improvement in evidence-based medicine curricula and continuous professional development to improve information literacy for better information retrieval and patient care.

## INTRODUCTION

Physicians frequently ask clinical questions during patient encounters at the point of care that require engaging in information seeking to identify and retrieve relevant evidence-based health-related information [1]. In practice, physicians encounter frequent barriers to retrieving relevant clinical information for patient care, including time during the encounter, lack of information searching skills, cost, and accessibility of knowledge resources [2–5]. In addition to information literacy and evidence-based medicine (EBM) skills, physicians report a lack of digital or Internet skills as a barrier to online health information retrieval [6].

Information and digital literacy are necessary for information retrieval satisfaction among healthcare professionals [7]. However, the perspective of digital literacy is still underused in published healthcare studies. A scoping review about digital health competencies for primary health care professionals yielded only 28 articles, with the majority published before 2011 and conducted in developed countries such as the U.S., U.K., Australia, Canada, and Europe, with one article from Malawi [8]. Moreover, only 20% of the articles focused on basic computer and information literacy skills, and the majority focused on using electronic medical records. Therefore, this study aims to measure the association between community family physicians' information retrieval and their information and computer literacy.

## MATERIALS AND METHODS

This study is a part of a cross-sectional, online, anonymous survey-based Ph.D. dissertation study that aimed to understand the characteristics of information retrieval of community family physicians at the point of care in developing countries and its predictors [9]. Community family physicians without academic affiliation in developing countries were targeted as their access to reliable resources or paid point-of-care resources and the Internet in the office may be limited [10–12]. Community family physicians without any academic affiliation were recruited by email invitation through the professional scientific societies of the World Organization of Family Doctors-Eastern Mediterranean Region (WONCA EMR) countries (Appendix 1). While it is not possible to know how many physicians received the email invitation, the population of interest's estimated size was 19,600 doctors [13]. The study was approved by the institutional review board (IRB) of the American University of Beirut. Appendix 2 includes the survey.

### Digital Literacy

There is no consensus definition or framework for digital literacy, and specific digital literacy skills differ among academic disciplines, such as education, information studies, or media studies, which can make it difficult to measure and assess across groups [14]. Digital literacy has been used as an umbrella term for different types of literacies: computer literacy, information literacy, network literacy, communication literacy, visual literacy, and technology literacy [15]. This study focused on two specific domains of digital literacy: information literacy and computer literacy [16].

Information literacy was measured using the Information Literacy Self-Efficacy-M scale [17]. It was adapted from the Information Literacy Self-Efficacy Scale developed by Kurbanoglu et al. [18] to include items relevant to the specific context of medical curricula. The total scale consisted of 5 subscales: evaluating and processing information (11 items), searching and finding information (10 items), medical information literacy (10 items), using the physical library (4 items), and bibliography (4 items). Another study used the scale to measure predictors of information literacy among medical students [19]. The internal consistency of the subscales was high, with Cronbach's alpha in the range of 0.858 to 0.930. The exploratory factor analysis of the five factors and 35 items accounted for 58.34% of the total variance. The total scale score was the sum of the various item responses, with higher scores indicating higher information literacy. Two relevant subscales were used for this research study: searching and finding information and medical information literacy. Furthermore, three questions in the subscales were removed as they are not relevant to the research question: finding citing authors, referencing the sources I use in a reference style used in medicine, and using different kinds of print sources (such as books, periodicals, encyclopedias).

Computer literacy was measured using the General Confidence With Computer Use Scale. It was first developed and validated within the context of learning mathematics among university students [20]. The scale was later validated among a sample of pharmacists in Lebanon [21]. It comprised 12 items answered using a 5-point Likert scale from 1 (strongly disagree) to 5 (strongly agree). The total score was the summation of the answers to all the questions. Higher scores indicated higher computer literacy. The internal consistency was good, with a Cronbach's alpha of 0.716. Using exploratory factor analysis, the 12 items explained 57.1% of the total variance.

### Statistical Analysis

Efficacy refers to the ability to complete tasks in order to achieve a desired result. Efficiency involves completing tasks with minimal expenditure of resources, such as time [22]. The efficacy and efficiency of respondents' information retrieval skills were examined with the questions “I find relevant information” and “it is easy to find the information,” respectively. One-way ANOVA was used to measure the association between the efficacy and efficiency of respondents' information retrieval skills and their computer literacy and information literacy, respectively. The data were analyzed with IBM SPSS Statistics (Version 27), and the significance level, α, was set at 0.05.

## RESULTS

A total of 72 participants from 8 countries were included in the analysis. Table 1 shows the demographics of the participants. Almost three-quarters of the participants (53/72, 73.6%) received formal training in EBM during their residency training. Almost two-thirds (44/72, 61.1%) attended a course or workshop on EBM [10].

**Table 1:** Demographics of the Surveyed Family Physicians

Demographic	*Median*	*25th-75^th^ percentile*
Age	38.0	28.0–69.0
Years of practice	6.0	3–14
Number of patients seen weekly at the clinic	50.0	27.5–100
	*n*	Percentage
Sex		
Females	41	61.2
Males	26	38.8
Country of practice		
Bahrain	3	4.5
Egypt	3	4.5
Iraq	21	31.8
Jordan	4	6.1
Kuwait	4	6.1
Lebanon	17	25.8
Saudi Arabia	9	12.5
United Arab Emirates	5	6.9
Location of Practice		
City	65	95.5
Suburban	2	2.9
Rural	1	1.5

Note. N = 72. Missing values exist.

The participants looked for digital clinical information at the point of care on average 14.0 times (SD = 34.4) per week with a median of 5.0 [25% percentile = 3, 775th percentile = 11.5]. Digital literacy was operationalized with two scales that measure information and computer literacy. As the two scales used for information and computer literacy were not validated in a similar population of family physicians, the internal consistency of the scales in the sample was studied using Cronbach's alpha. The total scale score is the sum of the various item responses, with higher scores indicating higher information or computer literacy for both scales. The mean total score for the information literacy scale was 59.8 (SD = 11.4), with a Cronbach alpha of 0.862. The most challenging aspects were using PICO (Patient/Population, Intervention, Comparison, Outcome) and MeSH (Medical Subject Headings) terms, using a factual database with the retrieval of articles and evaluating bias (Table 2). The maximum score was 91. The mean total score was 29.3 (SD = 5.6) for the computer literacy scale, with a Cronbach alpha of 0.710. The maximum score was 55.

**Table 2:** Information Literacy scale

I feel confident and competent to:	1–3 (Almost never true, usually not true, sometime but infrequently true	4 Occasionally true	5–7 (Often true, usually true, always true)
	n (%)		
Medical information literacy skills			
Initiate search strategies by using keywords and Boolean logic	13(18.1)	14(19.4)	45(62.5)
Use PICO	28(38.8)	25(34.7)	19(26.4)
Search for EBM information	12(16.7)	14(19.4)	46(64.0)
Use a factual database	18(25.0)	22(30.6)	32(44.4)
Use MeSH	32(44.4)	17(23.6)	23(32.0)
Use PubMed	10(13.9)	27(37.5)	35(48.6)
Retrieve an article of an institutional repository	19(26.4)	25(34.7)	28(38.9)
Evaluate bias	24(33.3)	27(37.5)	21(29.2)
Searching and finding information			
Define the information I need	6(8.3)	16(33.3)	50(69.4)
Decide where and how to find the information I need	7(9.7)	12(16.7)	53(73.6)
Identify a variety of potential sources of information	(13.9)	19(26.4)	43(60.0)
Use electronic information sources	5(7.0)	9(12.5)	58(80.6)
Use internet search tools (search engines, directories)	11(15.3)	11(15.3)	50(69.4)

A one-way ANOVA revealed a statistically significant difference between information retrieval efficacy and information literacy between at least two groups (F (2,69) = 4.466, p = 0.015). The efficiency of information retrieval was also associated with information literacy (F (2.69) = 4.563, p = 0.014). Table 3 shows the Bonferroni test for multiple comparisons. Computer literacy was not associated with information retrieval efficacy or efficiency.

**Table 3:** Association Between Efficacy/Efficiency of Information Retrieval and Information and Computer Literacy (N = 72)

	It is easy to find the information (efficiency) M (S.D.)				I find relevant information (efficacy) M (S.D.)			
	*Always*	*Often*	*Sometimes*	*p*-value*	*Always*	*Often*	*Sometimes*	*p*-value*
Computer Literacy	30.6(8,1)	29.2(4.0)	28.3(6.5)	0.516	30.2(7.0)	28.9(4.3)	28.0(7.5)	0.5 65
Information Literacy	62.6(12.3)^a^	61.5(10.8)^b^	52.7(10.0)^a^^b^	0.014	64.5(12.1)	57.4(10.3)	52.2 (8.7)	0.015

* One-way ANOVA;

a p-value = 0.038;

b p-value = 0.21

## DISCUSSION

The efficiency of information retrieval, lack of information retrieval abilities, and ease of use have been reported by physicians as barriers to information retrieval [2–5]. Information and digital literacy are necessary for information retrieval satisfaction among healthcare professionals [7]. This study aimed to understand the relationship between information retrieval efficacy and efficiency with digital literacy. The computer and digital literacy scores of the community physicians in the 8 Arab countries were average. Information literacy, rather than computer literacy, was associated with the efficacy and efficiency of information retrieval behavior.

Digital literacy among healthcare professionals and practicing physicians, in particular, is still understudied [8]. In this study, community family physicians scored average on information and computer literacy scales. A sample of primary health care physicians in KSA scored 12.84±4.4 out of 24 on their level of knowledge about extracting journals and databases relevant to EBM [23]. A study among nursing students in Australia has shown good competency in basic computer and information literacy skills; however, they were less competent in translating the skills in a clinical context [24]. Similarly, physicians in seven hospitals in KSA scored 80-90% on a 22-item questionnaire regarding their basic computer skills [25]. A cross-sectional study among a diverse group of healthcare professionals in five teaching hospitals in Iran has shown high scores of 83.9% and 80.6% on operational skills and information searching, respectively [26]. A systematic review of pharmacy staff digital literacy levels conducted in 2016 has identified only three studies that lack quantifiable, measurable data on digital literacy [27]. 65.9% of physicians in Wuhan, China, have reported no or little EBP competence regarding medical information retrieval [28]. Family physicians reported a lack of digital and computer skills among major barriers to online health information retrieval [6]. This study is novel in objectively measuring physicians' digital literacy using validated tools. The need to quantify the digital literacy levels of physicians is of utmost importance to better understand the current situations and propose further research exploring the predictors of digital literacy.

This study suggests that information literacy, rather than computer literacy, is associated with better information retrieval efficacy and efficiency among health care professionals. Therefore, there is a need for improvement in the current curricula. EBM has been incorporated into the medical curricula of undergraduate medical students, postgraduate and practicing physicians. However, critical appraisal (Step 3 in evidence-based practice) was the most frequently taught skill, and there was less focus on teaching search strategies and information retrieval skills [29]. The same theme was found in a thematic systematic review of evidence-based practice nursing education, where the focus on critical thinking and analysis was emphasized [30]. Medical librarians consider that information literacy instruction should be mandatory for medical students and delivered through workshops in coordination with medical faculty [31]. A scenario-based workshop delivered by clinical educators and the medical librarian was proven effective by the participants regarding engagement, satisfaction, and reported benefits in actual clinical practice [32]. EBM training resulted in a short-term improvement of knowledge; however, there is a lack of studies that support long-term skills retention beyond one year [33]. There may be a gap between what we teach and what physicians practice in real life, especially with the new surge of summary databases and point-of-care decision tools. Collaboration and communication among faculty, librarians, and students are needed to better understand physicians' current practices and develop educational programs to improve students' and practicing physicians' information literacy skills through interactive learning activities [30, 34].

## LIMITATIONS OF THE STUDY

No unique definition of digital literacy has been adopted in published studies [35–38]. There was no clear distinction between information, computer, and digital literacy [39]. The validated tools used to measure digital literacy are self-reported surveys where people tend to over-report their abilities. Further research could use vignette-based designs to objectively measure the efficacy and efficiency of information retrieval.

## CONCLUSION

Community family physicians scored average on self-reported information and computer literacy scales. Information retrieval at point of care efficacy and efficiency were associated with information literacy rather than computer literacy. EBM curricula should be modified to develop information literacy among healthcare professionals for better information retrieval at the point of care and, consequently, better patient care.

## Data Availability

Data available on request from the authors.

## DECLARATIONS

Ethical approval and consent to participate: The study was approved by the Institution Review of Board of the American University of Beirut and Walden University.

Consent for publication: Not applicable.

Availability of data and material: All data generated or analyzed during this study are included in this published article.

Disclosure of interest: The authors report there are no competing interests to declare.

Funding: This research did not receive any specific grant from funding agencies in the public, commercial, or nonprofit sectors.

Patient and public involvement: Neither the patients nor the public was involved in the research design.

Acknowledgments: None.

Biographical note: Dr. Jumana Antoun is a medical education and core faculty at a family medicine residency, teaching evidence-based medicine. Dr. Jennifer Lapin has experience in medical education research, program evaluation, and psychometrics. Dr. Dennis Beck is an educational technology professor interested in digital equity and immersive learning environments.

## SUPPLEMENTAL FILES

**Appendix A:** List of the Family Medicine Services

**Appendix B:** Online Survey
