# Supplementary material for: Efficacy and efficiency of information retrieval of community family physicians at the point of care: exploring the associations with information and computer literacy
Source: J Med Libr Assoc. 2023 Jul 10;111(3):677–83. doi: 10.5195/jmla.2023.1539 (PMC10361562; doi:10.5195/jmla.2023.1539)
Supplement: Supplementary file 1 — Appendix A: List of the Family Medicine Services [file jmla-111-3-677-s01.pdf]

Appendix A: List of the Family Medicine Societies

Algerian Society of General Medicine / Societe Algerienne De Medecine Generale (SAMG)

Bahrain Family Physicians Association

Egyptian Family Medicine Association (EFMA)

Emirates Family Medicine Society (EFMS)

Iraqi Family Physicians Society (IFPS)

Jordan Society of Family Medicine (JSFM)

Kuwaiti Association of Family Physicians and General Practitioners (KSFGP)

Lebanese Society of Family Medicine (LSFM)

National Collective of General Practitioners of Marocco (MG Maroc)

Oman Family and Community Medicine Society

Palestinian Association of Family Medicine (PAFM)

Saudi Society of Family and Community Medicine (SSFCM)
