# Supplementary material for: Efficacy and efficiency of information retrieval of community family physicians at the point of care: exploring the associations with information and computer literacy
Source: J Med Libr Assoc. 2023 Jul 10;111(3):677–83. doi: 10.5195/jmla.2023.1539 (PMC10361562; doi:10.5195/jmla.2023.1539)
Supplement: Supplementary file 2 — Appendix B: Online Survey [file jmla-111-3-677-s02.pdf]

Appendix B: Online Survey

Are you a family physician who practices in the community without any academic affiliation? Yes No

If no, the survey will end and the participant will be thanked
If yes, the survey will continue

Kindly note, throughout the questionnaire, **point of care** is defined as the time during or directly after the clinical encounter with the patient in the clinic

General Questions

1. Age _____ years
2. Sex — 1) Female 2) Male

3. Country: ________________
4. Year of practice since graduation as family medicine: ___ years

5. Location of practice — 1) City 2) Suburban 3) Rural

6. Type of practice — 1) Solo practice 2) Group practice 3) Employed physician

7. What is the number of patients seen on weekly basis at the clinic? ______________

8. Country of Practice __________

Professional EBM Competencies

9. Have you received any formal training in evidence-based medicine during your residency? — 1) Yes 2) No
10. Have you ever attended a course or workshop on Evidence-Based Medicine? — 1) Yes 2) No

Digital Information Practice

11. On average, how many times per week do you look for digital clinical information at the point of care? _________ week

(This can be from search engines or online databases or mobile applications)

12. How would you rate your ability to find the information you require to answer clinical questions for patient care at the point of care?
    1) Very good 2) Good 3) Average 4) Poor 5) Very poor

13. How often do you need information **at the point of care** for the following reasons?

	Always	Often	Sometimes	Rarely	Never
Making diagnosis/workup plan					
Making a clinical decision concerning treatment options					
Medications (side effects, dosages, interaction)					
Providing information to patients					

14. How often did you look for medical information from the following sources **at the point of care**?

	Always	Often	Sometimes	Rarely	Never
Textbooks					
Clinical practice guidelines					
Online databases like Medline or Pubmed					
Subscribed online databases like UpToDate, Dynamed, Clinical Key					
Medical websites like Medscape or e-medicine					
General databases like Google or Google Scholar					
Medical apps like Epocrates, Medical calculator					
Peers/colleagues					
Pharmaceutical representatives					

15. List the top 3 digital information resources that you consult most often when your require information at point of care?

    ____
    ____
    ____

16. What is your first resource of information in general at point of care?

    ____
    ____

17. At the point of care, I search for digital medical information using:
    1) Computer 2) Mobile phone 3) Tablet

18. Do you have access to the internet at the point of care?
    1) Always 2) Often 3) Sometimes 4) Rarely 5) Never

19. I own one or more mobile apps for information retrieval at the point of care.
    1) Yes 2) No

20. If yes, how many apps: ______
    Please specify the top 3 frequent apps:

____
____

21. Do you own medical databases/apps that require a subscription?
    1) Yes 2) No

Information literacy

22. I feel confident and competent to:

	1 Almost never true	2 Usually not true	3 Sometimes but infrequently true	4 Occasionally true	5 Often true	6 Usually true	7 Always true
**Medical information literacy skills**							
Initiate search strategies by using keywords and Boolean logic							
Use PICO							
Search for EBM information							
Use a factual database							
Use mesh							
Use PubMed							
Retrieve an article of an institutional repository							
Evaluate bias							
**Searching and finding information**							
Define the information I need							
Decide where and how to find the information I need							
Identify a variety of potential sources of information							
Use electronic information sources							
Use internet search tools (search engines, directories)							

Computer literacy

21. The following statements refer to your confidence when using computers

	Strongly agree	Agree	Neutral	Disagree	Strongly disagree
I have less trouble learning how to use a computer than I do learning other things.					
When I have difficulties using a computer I know I can handle them.					
I am not what I would call a computer person.					
It takes me much longer to understand how to use computers than the average person. I have never felt myself able to learn how to use computers.					
I enjoy trying new things on a computer.					
I find having to use computers frightening.					
I find many aspects of using computers interesting and challenging.					
I don't understand how some people can seem to enjoy spending so much time using computers.					
I have never been very excited about using computers.					
I find using computers confusing.					

Technology Affordances

22. Please select the best answer concerning finding the clinical information that you need to answer clinical questions at the point of care

	Always	Often	Sometimes	Rarely	Never
I find relevant information					
I find useful information					
I find reliable unbiased information					
It is easy to find the information					
I find the information in less than 5 minutes					
I find the information in less than 2 minutes					
I am confident about the information that I find					
